# New Rapid Diagnostic Tests for *Neisseria* *meningitidis* Serogroups A, W135, C, and Y

**DOI:** 10.1371/journal.pmed.0030337

**Published:** 2006-09-05

**Authors:** Suzanne Chanteau, Sylvie Dartevelle, Ali Elhadj Mahamane, Saacou Djibo, Pascal Boisier, Farida Nato

**Affiliations:** 1 Institut Pasteur, Plateforme Technologique PT5, Paris, France; 2 CERMES, National Reference Center for Meningitis, Ministry of Health, Réseau International Instituts Pasteur, Niamey, Niger; University of London, United Kingdom

## Abstract

**Background:**

Outbreaks of meningococcal meningitis (meningitis caused by Neisseria meningitidis) are a major public health concern in the African “meningitis belt,” which includes 21 countries from Senegal to Ethiopia. Of the several species that can cause meningitis, N. meningitidis is the most important cause of epidemics in this region. In choosing the appropriate vaccine, accurate N. meningitidis serogroup determination is key. To this end, we developed and evaluated two duplex rapid diagnostic tests (RDTs) for detecting N. meningitidis polysaccharide (PS) antigens of several important serogroups.

**Methods and Findings:**

Mouse monoclonal IgG antibodies against N. meningitidis PS A, W135/Y, Y, and C were used to develop two immunochromatography duplex RDTs, RDT_1_ (to detect serogroups A and W135/Y) and RDT_2_ (to detect serogroups C and Y). Standards for Reporting of Diagnostic Accuracy criteria were used to determine diagnostic accuracy of RDTs on reference strains and cerebrospinal fluid (CSF) samples using culture and PCR, respectively, as reference tests. The cutoffs were 10^5^ cfu/ml for reference strains and 1 ng/ml for PS. Sensitivities and specificities were 100% for reference strains, and 93.8%–100% for CSF serogroups A, W135, and Y in CSF. For CSF serogroup A, the positive and negative likelihood ratios (± 95% confidence intervals [CIs]) were 31.867 (16.1–63.1) and 0.065 (0.04–0.104), respectively, and the diagnostic odds ratio (± 95% CI) was 492.9 (207.2–1,172.5). For CSF serogroups W135 and Y, the positive likelihood ratio was 159.6 (51.7–493.3) Both RDTs were equally reliable at 25 °C and 45 °C.

**Conclusions:**

These RDTs are important new bedside diagnostic tools for surveillance of meningococcus serogroups A and W135, the two serogroups that are responsible for major epidemics in Africa.

## Abstract 2

There are several strains of*Neisseria meningitidis* that can cause seasonal outbreaks of meningitis in Africa. Treatment of patients and containment of the epidemic through vaccination depends on which strain is responsible. The new dipstick tests described here are accurate and suitable for storage and use in resource-poor settings.

## Editors' Summary

**Background:**

Bacterial meningitis, a potentially deadly infection of tissues that line the brain and spinal cord, affects over 1 million people each year. Patients with bacterial meningitis usually have fever, headache, and stiff neck, and may become unconscious and die if the disease is not treated within hours. Most cases of bacterial meningitis occur in Africa, particularly in the arid savannah region south of the Sahara known as the Sahel, where epidemic outbreaks of meningitis occur periodically. This region, also called the “meningitis belt,” extends from Senegal and adjacent coastal countries in West Africa across the continent to Ethiopia. Although most outbreaks tend to occur in the dry season, they differ in frequency in different areas of the meningitis belt, and may involve any of several kinds of bacteria. One of the major causes of epidemic meningitis is *Neisseria meningitidis,* a meningococcus bacterium that exists in several different groups. Group A has been a common cause of epidemic meningitis in Africa, and some outbreaks were due to group C. More recently, group W135 has emerged as an epidemic strain. In addition to prompt diagnosis and treatment of individual cases, effective public health strategies for controlling meningococcal meningitis include rapid identification of outbreaks and determination of the type of bacteria involved, followed by mass vaccination of people in the surrounding area without delay. Vaccines are chosen on the basis of the responsible meningococcal serogroup: either the inexpensive bivalent vaccine A/C or the expensive, less readily available trivalent vaccine A/C/W135. Before the advent of W135 as an epidemic clone, bivalent vaccine was applied in the meningitis belt without identification of the serogroup. With the appearance of the W135 strain in 2003, however, the determination of serogroup before vaccination is important to select an effective vaccine and avoid misspending of limited funds.

**Why Was This Study Done?:**

Because there are few laboratories in the affected countries and epidemiological surveillance systems are inadequate, it is difficult for health authorities to mount a rapid and effective vaccination campaign in response to an outbreak. In addition, because the two main bacteria (meningococcus and pneumococcus) that cause meningitis require different antibiotic treatments, it is important for doctors to find out quickly which bacteria is causing an individual case. The authors of this study wanted to develop a rapid and easy test that can tell whether meningococcus is the cause of a particular case of meningitis, and if so, which group of meningococcus is involved. As most outbreaks in the meningitis belt occur in rural areas that are distant from well-equipped medical laboratories, it was necessary to develop a test that can be carried out at the patient's bedside by nurses, does not require refrigeration or laboratory equipment, and is highly accurate in distinguishing among the different groups of meningococcus.

**What Did the Researchers Do and Find?:**

The researchers have developed a rapid test to determine whether a patient's meningitis is caused by one of the four most common groups of meningococcus circulating in Africa. The test is done on the patient's spinal fluid, which is obtained by a lumbar puncture (spinal tap) as part of the usual evaluation of a patient thought to have meningitis. The test uses two paper strips, also called dipsticks (one for groups A and W135/Y, and the other for groups C and Y), that can be placed in two separate tubes of the patient's spinal fluid. After several minutes, the appearance of red lines on the dipsticks shows whether one of the four groups of meningococcus is present. The dipsticks can be produced in large quantities and relatively cheaply. The researchers showed that the test dipsticks are stable for weeks in hot weather, and are therefore practical for bedside use in resource-poor settings. They examined the test on stored spinal fluid from patients in Niger and found that the dipstick test was able to identify the correct group of meningococcus more than 95% of the time for the three groups represented in these specimens (the results were compared to a standard DNA test or culture that are highly accurate for identifying the type of bacteria present but much more complicated and expensive).

**What Do These Findings Mean?:**

The new dipstick test for meningococcal meningitis represents a major advance for health-care workers in remote locations affected by meningitis epidemics. This test can be stored without refrigeration and used at bedside in the hot temperatures typical of the African savannah during the meningitis season. The dipsticks are easier to use than currently available test kits, give more rapid results, and are more accurate in telling the difference between group Y and the increasingly important group W135. Further research is needed to determine whether the test can be used with other clinical specimens (such as blood or urine), and whether the test is dependable for detecting group C meningococcus, which is common in Europe but rare in Africa. Nonetheless, the dipstick test promises to be an important tool for guiding individual treatment decisions as well as public health actions, including vaccine selection, against the perennial threat of epidemic meningitis.

**Additional Information.:**

Please access these Web sites via the online version of this summary at http://dx.doi.org/10.1371/journal.pmed.0030337.

- World Health Organization fact sheet on meningococcal meningitis
- PATH Meningitis Vaccine Project
- US Centers for Disease Control and Prevention page on meningococcal disease

## Introduction

It is estimated that annually over one million cases of meningitis are caused by three bacterial species: Neisseria meningitidis (also termed meningococci, the cause of meningococcal meningitis or disease), *Streptococcus pneumoniae,* and Haemophilus influenzae b [1]. Together, these organisms cause substantial morbidity and mortality globally. N. meningitidis is the cause of epidemic meningitis associated with high mortality worldwide, but more than 50% of all of these meningococcal meningitis cases are reported from sub-Saharan Africa's “meningitis belt,” which extends from Senegal to Ethiopia and includes 21 countries with a total at-risk population of 250 million [1]. Severe annual outbreaks of meningococcal meningitis are reported in this region, and incidence of the disease may be as high as 1,000 per 100,000 inhabitants [3–5]. Outbreaks occurring during the past ten years (1995–2004) have resulted in almost 700,000 cases and 60,000 deaths [5].

N. meningitidis polysaccharide (PS) serogroup A is responsible for major epidemics in Africa, but the recent change in epidemiological pattern due to the emergence of serogroup W135 as an epidemic strain is a cause of particular concern [6–8]. The WHO strategy for controlling epidemic meningococcal disease in this region is based on (i) early and accurate detection and (ii) rapid implementation of mass vaccination using an appropriate polysaccharide (PS) vaccine [9]. However, the response to epidemic meningococcal disease in Africa is hindered by delays in (or even the lack of) identification of the causal agent and by the scarcity and high cost of polyvalent vaccines. Decision-makers at country level in the meningitis belt need to make use of the best epidemiological and laboratory evidence for choosing the most appropriate vaccine. The implementation of alert and epidemic thresholds and of a decisional tree, based on reliable identification of the aetiological agent for selection of the most appropriate vaccine, is recommended [1,10].

The gold standard diagnostics for meningococci are the classic culture with subsequent identification of serogroup using specific antisera and the multiplex PCR method [11]. In the sub-Saharan countries, neither of these two tests is available outside the capitals, large cities, or reference laboratory settings [12]. The old nonculture tests (leucocyte cell count and Gram stain microscopy), although rapid and cheap, cannot identify the serogroup responsible for a given outbreak to guide the choice of vaccine. Currently, latex agglutination tests (LATs) are most widely used to identify meningococcal PS serogroups in the cerebrospinal fluid (CSF) of patients. The Pastorex (Bio-Rad) kit for detecting serogroup A, B, C, and W135/Y has been evaluated for serogroup A and W135 under reference laboratory conditions [13] and in a district laboratory in Niger [14]. The sensitivity and specificity are good, ranging from 84.9% to 88% and from 93% to 97.4%, respectively. This kit does not, however, differentiate between serogroups W135 and Y, and its performance under field conditions lacking basic laboratory equipment is not known. Furthermore, this kit requires cold storage, and its high cost does not allow its use in every district or health care centre. Together these difficulties explain the consistent failure of outbreak aetiology identification and the lack of proper case management (resulting in inadequate antimicrobial treatment), which are main causes of death due to meningococcal disease.

In view of this situation, we therefore aimed to develop and evaluate the diagnostic accuracy of easy-to-perform rapid diagnostic tests (RDTs) for the specific and sensitive detection of meningococcal PS serogroup antigens that can be used in resource-poor countries, especially near rural populations and at the patient's bedside.

## Methods

### Standard Diagnostic Methods

Culture and PCR were used as gold-standard diagnostic methods. They were performed by two trained technicians according to reference techniques routinely used at the CERMES (National Reference Centre for Meningitis, Niamey, Niger) [12].

For culture, CSF specimens were drawn from clinically suspected cases of meningitis according to the national guidelines of the Ministry of Health. The specimens were cultured on blood agar and chocolate agar (at 37 °C with 5% CO_2_) and the serogroups of the N. meningitidis isolates were determined by agglutination with serogroup-specific antisera (Bio-Rad, Marnes-la-Coquette, France, and DIFCO, Detroit, Michigan, United States).

Multiplex PCR was performed on freeze-boiled CSF samples to amplify the *crgA* gene of N. meningitidis [11], the *lytA* gene of S. pneumoniae [15], and the *bexA* gene of *H. influenzae* [16]*.* For genogrouping of meningococcus-positive specimens, a second PCR was performed to amplify the *siaD* gene for serogroups B, C, Y, and W135 and the *orf-2* of the *mynB* gene for serogroup A [11]. Specimens testing positive for H. influenzae type b were confirmed by amplification of the *cap* gene [16]. PCR was performed in a Primus 96 thermocycler (MWG Biotech, Ebersberg, Germany). The detection threshold for PCR was 1–10 bacteria per assay, as shown by CSF samples spiked with serial 10-fold dilutions of reference strain suspensions of N. meningitidis serogroups A, B, C, Y and W135, *S. pneumoniae,* and H. influenzae b.

A “negative” CSF sample was negative by culture and PCR for *N. meningitidis, S. pneumoniae,* and H. influenzae. A “confirmed” CSF sample was a specimen that tested positive by culture and/or PCR for *N. meningitidis, S. pneumoniae,* or H. influenzae.

### Development of RDTs for the Detection of Meningococcal PS Antigen

Anti-N. meningitidis monoclonal antibodies (Mabs) were developed at the Institut Pasteur (Paris, France). Biozzi BP/2 mice were immunized with four different PS conjugates (A, W135, C, and Y). Conventional fusion and ELISA screening of resulting hybridomas were carried out and IgG Mabs were selected on the basis of high specificity and affinity for purified A, W135, C, and Y PS. Conjugates and PS were kindly provided by Sanofi Pasteur. The selected Mabs were: K15–2 (anti-serogroup A IgG1, kappa chain); B6–1 (anti-serogroup C IgG1, kappa chain); L14–7 (anti-serogroup Y IgG2b, kappa chain); and C18–3 (anti-serogroup W135/Y IgG1, kappa chain). Although we obtained Mab D20–19, which is specific to serogroup W135 by ELISA, we did not use it because it cross-reacts with serogroup Y when used in immmunochromatography. The four selected Mabs were conjugated to gold particles by British Biocell International (Cardiff, UK), and four monovalent one-step vertical flow immunochromatography dipsticks (N. meningitidis serogroups A, C, Y, and W135/Y) were designed and optimized as previously described [17,18], with Accuflow polyester (Schleicher & Schuell Bioscience, Ecquevilly, France) used as the conjugate pad [19]. Homologous Mabs directed against serogroups A, C, Y, and W135/Y were sprayed onto the nitrocellulose at a high concentration of 2 μg per centimeter line, to give a specific capture line. As a control line for the capture of gold particles conjugated with mouse Mab, goat anti-mouse IgG was sprayed onto the nitrocellulose at a concentration of 0.8 μg/cm (ICN Biomedicals, Aurora, Ohio, United States). The hybridomas have been deposited at the Collection Nationale des Microorganismes (Institut Pasteur, Paris, France) and are available upon request to Institut Pasteur.

**Figure 1:**
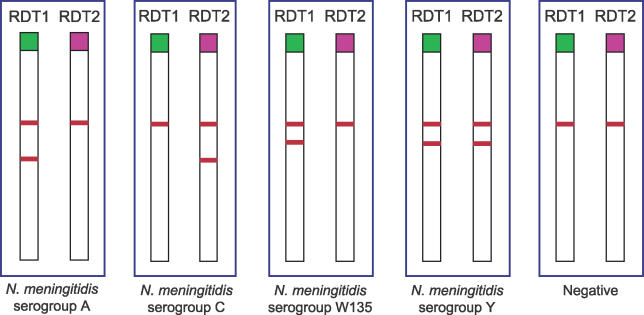
RDT Results by Serogroup Results of RDT_1_ and RDT_2_ are shown for each N. meningitidis serogroup.

Four monovalent dipsticks (serogroups A, W135/Y [i.e., detects both W135 and Y but does not distinguish between them], C, and Y) were optimized and preliminary tests carried out. They were assembled as two duplex dipsticks—RDT_1_ for A and W135/Y and RDT_2_ for C and Y (see Figure 1)—for evaluation by CERMES. The RDTs were stored at 4 °C in a moisture-proof bag until use. The test was carried out in 3 ml disposable plastic tubes at 25 °C (air-conditioned laboratory), and a volume of 150–200 μl of plain CSF or reference strain suspension in PBS, pH 7.2 was used. After incubation of the dipstick with its end in the sample for 10–15 min, the chromatographic result was recorded. A negative result consisted of a single upper pink control line, and a positive result consisted of two pink lines, the upper control line and the lower positive line.

### Evaluation of Duplex RDTs

#### Sensitivity and specificity.

The sensitivity and specificity of each RDT were assessed by one trained technician.

Reference isolates of N. meningitidis (serogroups A, B, C, W135, Y, and X), *N. lactamica, S. pneumoniae,* and H. influenzae b were obtained from the 2003–2004 collection of strains of CERMES and from the collection of the WHO Collaborating Centre for meningococci (IMTSSA, Marseille, France) and stored at −80 °C in brain-heart infusion supplemented with 10% glycerol. The bacteria were cultured on blood agar at 37 °C with 5% CO_2_ for 16 h. The colonies were suspended in PBS, adjusted to a concentration of 10^8^ cfu/ml (colony count of serial dilutions plated on blood agar), and heat-killed for 2 h at 60 °C. Numbers and reference strains tested for specificity were: 30 N. meningitidis of serogroup W135, 25 serogroup Y, 18 serogroup A, ten serogroup X, 11 serogroup B, seven serogroup C, nine *N. lactamica,* ten *S. pneumoniae,* and ten H. influenzae b. Numbers and strains tested for sensitivities were: ten N. meningitidis of serogroup A, 30 serogroup W135, 25 serogroup Y, and seven serogroup C.

CSF samples were collected in 2003–2005 from patients in Niger, before treatment, who were documented as suspected of having meningitis; these samples were tested by the reference standard tests (culture and/or multiplex PCR). These samples were stored at −20 °C until use. Numbers and strains tested for specificities were: 255 *N. meningitidis* of serogroup A, 48 serogroup W135, 11 serogroup X, 30 *S. pneumoniae,* 21 H. influenzae b, and 162 negative for *N. meningitidis/S. pneumoniae/H. influenzae* b; and for sensitivities 255 N. meningitidis of serogroup A, 48 serogroup W135, and four serogroup Y. No CSF of serogroup C was available.

#### Cutoff, reproducibility, and shelf life.

The detection limit (cutoff) of each RDT was determined on 10-fold dilutions of reference N. meningitidis PS from the National Institute for Biological Standards and Control (Hertfordshire, UK) for serogroup C code 38/730, serogroup A code 98/722, serogroup Y code 01/426, and serogroup W135 code 01/428, and for calibrated reference meningococcal strains from the Manchester Medical Microbiology Partnership (Manchester, UK) serogroup C code 11, serogroup A code 243594, and serogroup W135 code M01.240070 and from CERMES, serogroup Y code 477–03. Meningococcal strains were added to and diluted in pooled negative CSF samples.

The reproducibility of the cutoffs was assessed by testing, ten times simultaneously and using the same batch of RDTs, calibrated suspensions (10^5^ cfu/ml) of the reference meningococcal serogroups A, W135, C, and Y.

To predict the shelf-life of our RDTs, we used the accelerated stability method that consisted in storing the assays for a time at elevated temperature [20]. RDT_1_ and RDT_2_ were tested over a period of three weeks of exposure at high temperature of 60 °C on serial dilutions of PS serogroup A, W135, C and Y, twice per week. This accelerated stability method is equivalent to two years of actual storage time at room temperature (25 °C) [17]. In addition, as these RDTs are intended to be used in the field outside of air-conditioned settings, we compared the reliability of the tests performed at 25 °C (air-conditioned room) and 45 °C (incubator), simulating normal room temperature in the Sahel during the meningitis season.

### Prospective Study

We compared the results obtained with PCR and RDT_1_ performed in a blind study by two different technicians, on 57 frozen CSF samples from patients with suspected meningitis. These samples were collected, before treatment, from patients in the Niamey and Maradi regions, from January 15th to February 11th 2005 and were received at the CERMES for aetiological diagnosis.

### Statistics

The evaluation was performed according to the STARD (Standards for Reporting of Diagnostic Accuracy) for new assays [21].

We calculated the sensitivity (Se) of the RDTs, which is the proportion of specimens with the target disorder in which the test result is positive; and the specificity (Sp), which is the proportion of specimens without the target disorder in which the test result is negative. The 95% confidence intervals (CIs) for Se and Sp were calculated [22].

We also calculated likelihood ratios (LR). The positive LR (LR+ = Se/[1 − Sp]) indicates how many times a positive result is more likely to be observed in specimens with the target disorder than in those without the target disorder. The negative LR (LR− = [1 − Se]/Sp) indicates how many times a negative result is more likely to be observed in specimens with the target disorder than in those without the target disorder. The test is more accurate the more LR differs from 1. LR+ above 10 and LR− below 0.1 were considered convincing diagnostic evidence [23]. The 95% CIs were calculated for LR+ and LR− [24]. LRs were not determined for a Se or Sp of 100%.

The diagnostic odds ratio (DOR), defined as the ratio of the odds of positive test results in specimens with the target disorder relative to the odds of positive test results in specimens without the target disorder, was calculated as follows [25]:

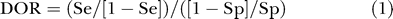



The DOR does not depend on prevalence and its value ranges from 0 to infinity, with higher values indicating better discriminatory test performance. The 95% CIs for DOR values were calculated [26].

The positive predictive value (PPV) represents the proportion of test-positive specimens that truly present the target disorder, while the negative predictive value (NPV) represents the proportion of test-negative specimens that truly do not present the target disorder:

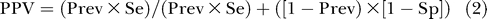
and

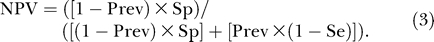



“Prev” is the prevalence of the target disorder in the population of specimens to which the test is applied. The 95% CIs for PPVs and NPVs were calculated [22].

Finally, we calculated the Cohen's kappa (κ) statistic [27] to measure concordance between PCR and RDT in the prospective blind study. κ may range from 0 to 1, and a κ value higher than 0.8 is thought to reflecting almost perfect agreement [28]. The 95% CIs for κ were calculated according to a method described by Fleiss [29].

### Ethical Aspects

All specimens were collected as part of the routine clinical management of patients, according to the national guidelines in Niger. Consequently, informed consent was not sought and approval from the national ethics committee was not required.

## Results

The diagnostic accuracy (based on STARD criteria) of the RDTs was assessed from December 2004 to November 2005, and was completed in February and March 2006.

### Cutoff and Reproducibility

As no monospecific Mab against W135 could be obtained, two duplex dipsticks were created: RDT_1_, for the detection of serogroups A and W135/Y, and RDT_2_, for the detection of serogroups C and Y and for discrimination between W135 and Y. The detection limits for the four serogroups were 1 ng PS/ml and 10^5^ cfu/ml of CSF. The cutoffs could be reproduced ten times for each of the four serogroups tested (A, W135, C, and Y), using calibrated suspensions (10^5^ cfu/ml) of reference strains.

No prozone phenomenon was observed with any of the RDTs at higher concentrations of PS (1 μg/ml) and bacteria (10^8^ cfu/ml).

### Sensitivities, Specificities, Likelihood Ratios, and Predictive Values

The sensitivities and specificities, with 95% CI, of the two RDTs with reference strains and documented CSF samples are summarized in Table 1. The sensitivity of RDT_2_ for CSF infected by serogroup C, a relatively rare serogroup in Africa, was not determined due to a lack of CSF samples but we were able to determine specificity. The specificity of the test for serogroup Y was 100%, but the sensitivity still must be accurately determined (all four CSF tested were positive).

**Table 1:**
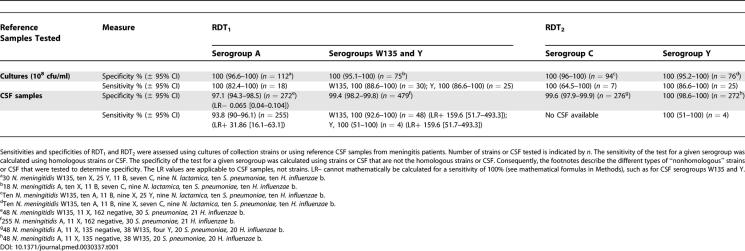
Validation of RDT_1_ (Serogroups A and W135/Y) and RDT_2_ (Serogroups C and Y) for the Diagnosis of Meningococcal Meningitis

For serogroup A identification, LR+ was 31.867 (95% CI, 16.1–63.1) and LR− was 0.065 (95% CI, 0.04–0.104), and the DOR was 492.9 (95% CI, 207.2–1,172.5). For serogroups W135 and Y, LR+ was 159.7 (95% CI, 51.7–493.3). It was not possible to calculate the other LRs and DORs because either their Se or their Sp was 100%.

**Figure 2:**
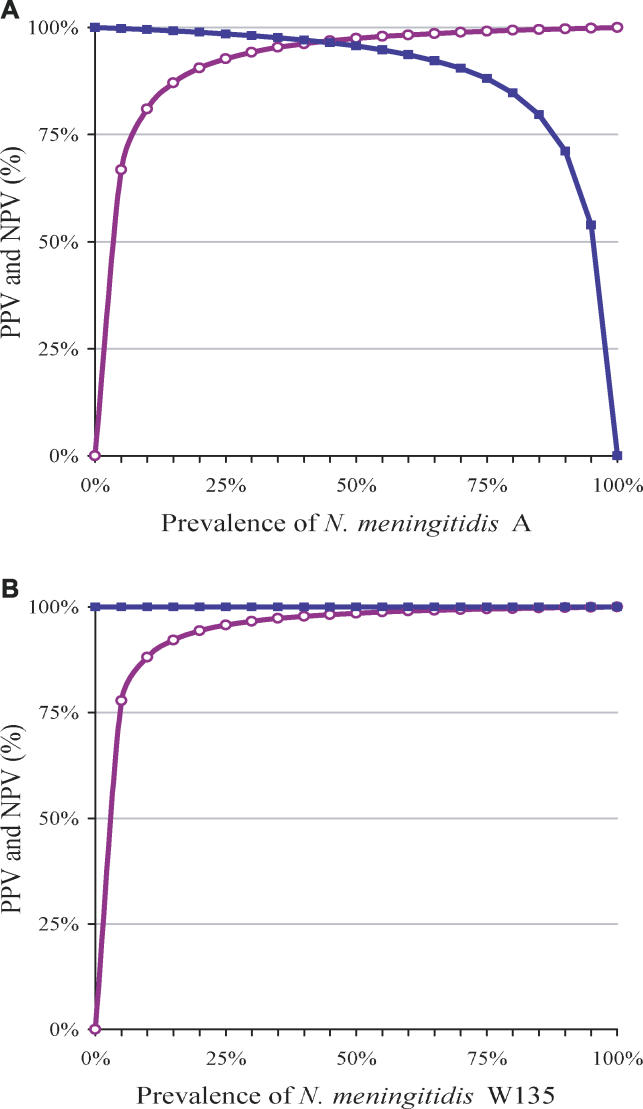
Predictive Values for N. meningitidis Diagnosis PVPs and NPVs for the diagnosis of N. meningitidis serogroup A (A) and serogroup W135 (B), according to prevalence. PVP is represented by the purple line with open circles, and NPV by the blue line with filled circles.

The variations of NPV and PPV for the diagnosis of serogroups A and W135, according to prevalence, were determined using the Se and Sp for clinical CSF samples (Figure 2). To diagnose the four serogroups (A, W135, C, and Y), we propose an algorithm based on the combination in series of RDT_1_ and RDT_2_. Figure 1 shows the results obtained for both tests on each serogroup.

### Shelf Life and Reliability

The results for RDT_1_ and RTD_2_ for the reference strains A, W135, and C were not affected by storage for 3 wk at 60 °C. For the serogroup Y, the test was stable for only 10 d at that temperature. RDT_1_ and RDT_2_ were equally accurate whether performed at ambient temperatures of 25 °C or 45 °C.

### Comparative Prospective Study

PCR tests on 57 CSF samples from patients with suspected bacterial meningitis received for aetiological diagnosis showed that 20 samples contained N. meningitidis (17 of serogroup A, two serogroup W135, and two serogroup X), 16 *S. pneumoniae,* four H. influenzae b; 16 samples tested negative (Table 2). RDT_1_ identified 17 samples as positive for N. meningitidis serogroup A, and two as positive for W135/Y. There were two discordant results (serogroup A), giving an overall concordance level of 96.5% (55/57) between the two tests, and a Cohen's κ coefficient of 0.92 (95% CI, 0.82–1.03).

**Table 2:**
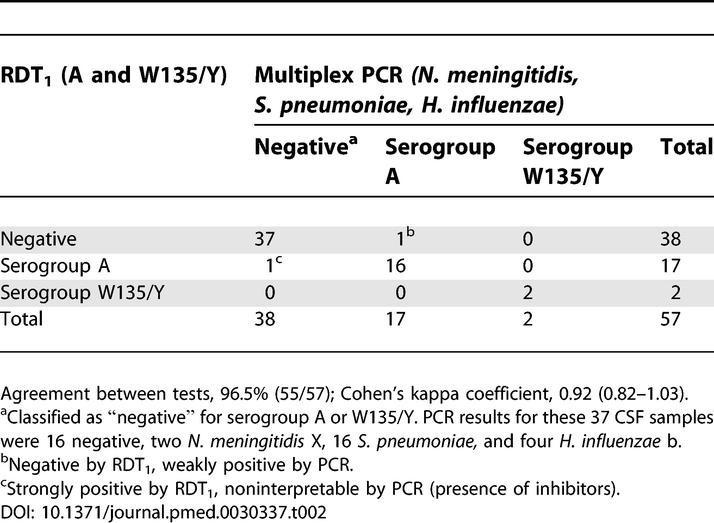
Comparative Results of RDT_1_ and PCR for CSF Samples from Patients with Suspected Meningitis

## Discussion

Because of the recent emergence of a new epidemic strain of *N. meningitidis,* serogroup W135, strengthening the capacity for epidemiologic and microbiologic surveillance and facilitating faster mass vaccination are urgently needed for effective prevention and control of epidemic meningococcal disease in Africa. A rapid diagnostic assay for the identification of meningococci is also needed to ensure administration of the appropriate antimicrobial treatment, to improve case management.

When hybridoma cell lines were screened by ELISA, one specific anti-W135 Mab was obtained; however, when assessed by immunochromatography (very high concentration of Mab on the nitrocellulose), it cross-reacted with serogroup Y, so it was not used. Previous IgG3 anti-W135 Mab has been described by Tsang and colleagues [30], but its specificity was assessed by ELISA and not by immunochromatography. In the present study, we developed and validated on reference bacterial strains two duplex dipsticks. The first, RDT_1_, detects PS antigens specific to meningococcal serogroups A and W135/Y. The second, RDT_2_, detects serogroups C and Y. The specificities and sensitivities for strains of the four serogroups were 100%. On CSF specimens from Niger, the specificities for serogroups A and W135 were excellent (97.1% and 99.3% respectively), as were the sensitivities (93.6% and 100%, respectively). Convincing diagnostic evidence is demonstrated for these two major and epidemic prone serogroups in Africa, by the positive and negative likelihood ratios of the tests.

The specificities of the RDTs for CSF infected with serogroups C and Y were excellent, but both serogroups are rare in Africa so we could not assess the sensitivity for serogroup C, because no CSF samples of patients infected with serogroup C were available; in addition, the sensitivity of the tests for serogroup Y still needs to be determined more accurately. Thus, usefulness of these RDTs is potentially limited for Europe or the U.S., where serogroups C and B are the main serogroups circulating. Further evaluation of the diagnostic accuracy of the RDT serogroup C will be conducted as well as a comparative study of both RDT_1_ and RDT_2_ in developed countries context. In the future, we plan to develop two other rapid tests for the detection of serogroup B, and serogroup X, an emerging threat in the African meningitis belt [5,31,32]**.**

The immunochromatography dipstick RDTs described here are simple to perform and can be used by health staff in the field. The detection limit of 1 ng PS/ml is similar to that of ELISA assays and lower than that of latex agglutination assays (10–100 ng PS/ml), explaining the higher specificities and sensitivities of RDTs compared with the Pastorex agglutination kit. In recent studies performed in Niger and Burkina Faso under reference laboratory conditions and in a district laboratory, the sensitivities and specificities of the Pastorex kit for the diagnosis of serogroups A and W135 were Se 84.9%–88% and Sp 93%–97.4% [13,14]. In contrast, the RDTs described in this study had sensitivities and specificities of, respectively, 93.8% and 97.1% for serogroup A and 100% and 99.4% for serogroup W135.

Both the Pastorex kit and the RDTs detect serogroup capsular PS antigen. The main advantages to detecting PS antigen are its large amount in the CSF and blood of meningitis patients and its stability to high temperature (even boiling). RDT_1_ (A and W135/Y) and RDT_2_ (C and Y) were as accurate and reliable at 25 °C as at 45 °C—the latter corresponding to typical room temperature during the meningitis season in the African meningitis belt. In most endemic areas, storage at temperatures above 25 °C for some length of time is unavoidable and should be taken into account. Preliminary stability data for RDT_1_ and RDT_2_ at 60 °C for 3 wk suggests that the tests could be stored at 25 °C for 2 y, or be transported to and stored at room temperature for some time in air-conditioned settings. Studies on improving the stability of RDT_2_ for serogroup Y are ongoing. These properties are of considerable importance for countries of the African meningitis belt where the ambient temperature ranges from 15 °C to 45 °C according to the season. To prevent degradation of RDTs on exposure to humidity during the rainy season, they should be individually packaged in moisture-proof envelopes that should remain sealed until immediately prior to use.

PCR and RDT_1_ tests gave concordant results in 96.5% of cases in a blind comparative study performed by two technicians. The kappa coefficient obtained in this study was high (0.92), reflecting almost perfect agreement. The two discordant results corresponded to two serogroup A CSF specimens: one was negative with RDT_1_ but weakly positive with PCR, and the second was strongly positive with RDT_1_ but uninterpretable with PCR, due to the presence of inhibitors (no amplification of the positive control DNA). A large-scale study comparing PCR and RDTs is warranted in the future, since PCR detects DNA and RDTs detect PS antigen. Thus RDTs may actually be more sensitive than PCR in patients with prior antibiotic therapy, sample contamination, delays in processing, or presence of PCR inhibitors.

Our RDTs are easier to perform than currently available latex agglutination kits. They also give more rapid results and discriminate more accurately between serogroups Y and W135, using the second test (RDT_2_) as a control. The negative and positive predictive values for the two main serogroups involved in outbreaks in the meningitis belt (A and W135) exceeded 95%, even for low prevalence of the disease. The RDTs merit evaluation (i) with other types of clinical samples (blood, urine) and on CSF samples of all serogroups from patients from developed countries; (ii) in operational field conditions of health care centres in Africa during epidemic and endemic periods; and (iii) on blood specimens of patients undergoing antibiotic therapy. These tests represent a major breakthrough for individual diagnosis and for surveillance of meningococcal diseases in the African meningitis belt that affect such a large proportion of meningitis patients in the world. The development of RDTs for serogroup B and X is ongoing, as well as for S. pneumoniae and H. influenzae b.

In order to make these RDTs available for supporting further field evaluation and epidemiological research, in-house small-scale production is being implemented in CERMES.

## Supporting Information

Alternative Language Abstract S1 French Translation of the Abstract (22 KB DOC)

Alternative Language Abstract S2 Spanish Translation of the Abstract (24 KB DOC)

## Abbreviations

CI: confidence interval
DOR: diagnostic odds ratio
LR: likelihood ratio
LR+: positive LR
LR−: negative LR
Mab: monoclonal antibody
NPV: negative predictive value
PPV: positive predictive value
PS: polysaccharide
RDT: rapid diagnostic test
Se: sensitivity
Sp: specificity
