# Supplementary material for: New Rapid Diagnostic Tests for Neisseria meningitidis Serogroups A, W135, C, and Y
Source: PLoS Med. 2006 Sep 5;3(9):e337. doi: 10.1371/journal.pmed.0030337 (PMC1563501; doi:10.1371/journal.pmed.0030337)
Supplement: Supplementary file 1 [file pmed.0030337.sd001.doc]

**Nouveaux tests de diagnostic rapide de**

***Neisseria meningitidis* sérogroupes A, W135, C et Y.**

**Résumé**

***Contexte*** : Les épidémies de méningite à méningocoques posent un problème important de santé publique dans les pays dit de la « ceinture africaine de la méningite ». Le choix du vaccin A/C bivalent ou multivalent incluant la valence W135, est tributaire de l’identification précise du sérogroupe responsable. Nous avons développé et évalué deux tests rapides duplex de diagnostic (RDTs) pour la détection de l’antigène capsulaire polysaccharidique (PS) du méningocoque.

***Méthodes/ Résultats principaux*** : des anticorps monoclonaux IgG anti- PS A, W135/Y, Y et C ont été utilisés pour mettre au point deux RDTs duplex sur le principe de l’immunochromatographie : RDT1 (sérogroupes A et W135/Y) et RDT2 (sérogroupes C et Y). La culture et la méthode PCR ont été adoptées comme tests de référence. Les critères STARD ont été appliqués pour déterminer la valeur diagnostique des RDTs sur des souches et des liquides céphalo-rachidiens (LCR) de référence.

Les seuils de détection pour tous les sérogroupes sont de 1ng PS /ml et 10^5^ cfu/ml. Les sensibilités et spécificités sont de 100% pour les souches de référence, et varient de 93,8 à 100% pour les sérogroupes A, W135 et Y. Pour le sérogroupe A, les « likelihood ratio » positif et négatif sont respectivement de 31.867 [16.1-63.1] et 0.065 [0.04-0.104], et le « diagnostic odds ratio » de 492.9 [207.2-1172.5]. Pour les sérogroupes W135 et Y, leur « likelihood ratio » positif est de 159.7. Les RDTs sont aussi fiables à 25°C qu’à 45°C.

***Conclusions/signification*** : ces RDTs sont de nouveaux outils importants et utilisables au chevet du malade pour la surveillance des méningocoques sérogroupes A et W135, les plus impliqués dans les épidémies en Afrique.

**Mots clés** : *Neisseria meningitidis*, diagnostic rapide, détermination sérogroupe, conservation, ceinture africaine méningite.
