# Supplementary material for: New Rapid Diagnostic Tests for Neisseria meningitidis Serogroups A, W135, C, and Y
Source: PLoS Med. 2006 Sep 5;3(9):e337. doi: 10.1371/journal.pmed.0030337 (PMC1563501; doi:10.1371/journal.pmed.0030337)
Supplement: Supplementary file 2 [file pmed.0030337.sd002.doc]

**Nueva Prueba Diagnóstica Rápida para los Serogrupos**

***Neisseria meningitidis* A, W_135_, C e Y**

**Sinópsis**

**Antecedentes**: La abrupta aparición de meningitis meningococcal es un problema de salud pública de alto riesgo, el cual se presenta en grandees niveles en el cinturón de meningitis Africano. La decisión de utilizar ya sea una vacuna bivalente A/C o multivalente a la cual se incluya W_135,_ depende en la precisión que se tiene para la determinación del serogrupo. En nuestro caso, hemos desarrollado y puesto a evaluación dos pruebas duplex de diagnosis rápida (RDTs) altamente exactos para la detección del antígeno polisacárido meningococcal (PS).

**Metodología/Hallazgos Principales**: Los anticuerpos monoclonales de ratón IgG en contra del PS A, W_135_/Y, Y y C fueron utilizados con el propósito de desarrollar dos duplex de inmunocromatografía RDTs, RDT_1_ (serogrupos A y W_135_/Y) y RDT_2_ (serogrupos C e Y). Siguiendo los criterios STARD, se logró determinar la exactitud de diagnosis de los RDTs, en cultivos de referencia y muestras de fluído cerebro-espinal (CSF), utilizando cultivos y PCR como muestras de referencia.

Los límites expuestos fueron 1ng PS/mL y 10^5^ cfu/mL. Las specificidad y grados de sensitividad fueron del 100% para las cultivos, con variaciones del 93.8 al 100% para los CSF serogrupos A, W_135_ e Y. Para el serogrupo A, los positivo y negativo “likelihood ratio” fueron de 31.867 [16.1-63.1] y 0.065 [0.04-0.104] respectivamente, y el “diagnostic odds ratio” fue de 492.9 [207.2-1172.5]. Para los serogrupos W_135_ and Y, el positivo “likelihood ratio” fue de 159.7. El RDT_s_ mostró la misma veracidad a temperaturas de 25°C y 45°C.

**Conclusiones/Significado**: Los presentes RDTs son nuevas e importantes herramientas de diagnóstico clínico para la observación de serogrupos meningococci A y W_135_, serogrupos que presentan una mayor vulnerabilidad a epidemias en Africa.

**Palabras claves**: *Neisseria meningitidis*, diagnósis rapida, determinación de serogrupos, cinturón Africano de meningitis.
